# Interleukin-4 receptor alpha is still required after Th2 polarization for the maintenance and the recall of protective immunity to Nematode infection

**DOI:** 10.1371/journal.pntd.0005675

**Published:** 2017-06-26

**Authors:** Justin Komguep Nono, Hlumani Ndlovu, Nada Abdel Aziz, Thabo Mpotje, Lerato Hlaka, Frank Brombacher

**Affiliations:** 1 Cytokines and Diseases Group, International Centre for Genetic Engineering and Biotechnology, Cape Town Component, Cape Town, South Africa; 2 Division of Immunology, Health Science Faculty, University of Cape Town & Immunology of Infectious Disease Research Unit, South African Medical Research Council (SAMRC), Cape Town,South Africa; 3 The Medical Research Centre, Institute of Medical Research and Medicinal Plant Studies (IMPM), Ministry of Scientific Research and Innovation, Yaoundé, Cameroon; 4 Department of Integrative Biomedical Sciences, Health Sciences Faculty, University of Cape Town, Cape Town, South Africa; 5 Department of Chemistry, Faculty of Science, Cairo University, Giza, Egypt; University of Manchester, UNITED KINGDOM

## Abstract

There is currently no vaccine against parasitic nematodes and the knowledge on the mechanisms by which protective immunity against this class of parasites is achieved is continuously expanding. Nematode parasites trigger a host protective type 2 immune response via interleukin-4 receptor alpha (IL-4Rα). Despite this central role, it is not known whether IL-4Rα has a role in maintaining host type 2 immune responses following polarization. To determine the role of IL-4Rα after polarization, we used a recently established strain of rosaCreER^T2-/+^IL-4Rα^-/Lox^ mice where *il4rα* gene deletion can be temporally controlled. We show that sustained expression of IL-4Rα is required for the maintenance of type 2 immune responses and protective immunity following interruption after polarization with *Nippostrongylus brasiliensis* primary infection. Moreover, we show by temporal deletion of IL-4Rα prior to secondary infection with *N*. *brasiliensis* that signaling via this receptor drives more efficient recall of type 2 immune responses and clearance of the parasites. Together, this study demonstrates that sustained IL-4Rα mediated signaling is required for the maintenance of anti-nematode type 2 immune responses, describing a novel function for IL-4Rα that is distinct from its role in immune polarization.

## Author summary

Parasitic nematodes infect humans and livestock worldwide, affecting human fitness and food security. Despite decades of research, the development of vaccines against these pathogens has been largely unsuccessful. Since the initiation and maintenance of anti-nematode immune responses is paramount for the design of effective vaccines, a better understanding of the mechanisms governing such host protective immune responses is essential. The authors herein demonstrate that host signaling via interleukin-4 receptor alpha (IL-4Rα) is not only needed for the establishment of protective type 2 immune responses during primary infection of mice with the parasitic nematode *Nippostrongylus brasiliensis*, but also critical for the maintenance of such responses thereafter. The authors also demonstrate that the host resistance to re-infection with this parasitic nematode in previously infected mice is promoted by an intact IL-4Rα mediated signaling. These data suggest a novel role for IL-4Rα in the maintenance and recall of protective anti-nematode immune responses and imply a need for sustained IL-4Rα mediated signaling to achieve better vaccination efficiency against parasitic nematodes.

## Introduction

Gut nematodes infect close to 2 billion people worldwide, making it the most common class of human parasitic infections until date [1]. The diseases caused by this class of parasites can be deleterious with symptoms ranging from anemia, growth stunting and undernutrition to cognitive deficits [1]. Gut nematodes induce massive T helper 2 immune responses in their host during infection [2–6]. In return, Th2 cells abundantly produce interleukin-4 (IL-4) and IL-13, two interconnected cytokines, which tightly control the expulsion of gut-dwelling nematode worms [5,7,8].

Signaling by both IL-4 and IL-13 depends on the binding to heterodimeric receptor complexes containing the IL-4Rα [9,10]. Cytokine binding results in intracellular signaling pathways that activate the transcription factor GATA-3, the master switch for the development of Th2 immune responses [11,12]. IL-4Rα mediated signaling on B cells drives the production of type 2 antibody isotypes IgG1 and IgE [13] that are the immunological hallmarks of anti-nematode immunity [14]. A central role has also been defined for signaling via IL-4Rα in the expansion and activation of type 2 innate immune cells such as eosinophils [15], alternative activation of macrophages [16], group 2 innate lymphoid cells [17] and mucus production by goblet cells or lung airway epithelial cells [18]). These adaptive and innate immune components act in concert to efficiently drive host protection against gut-dwelling nematodes [4,6,19].

Beyond the development of anti-nematode type 2 immune responses, the maintenance of such responses in previously infected hosts is the key to an efficient anti-nematode vaccine. In general for Th2 immune responses, a critical role for IL-4 production during immune priming has been defined for the subsequent maintenance of the Th2 phenotype [20]. Conversely, however, an IL-4-independent pathway has been demonstrated for the maintenance of memory Th2 cells *in vivo* where histone methylation of the IL-13 and IL-4 gene loci was fully preserved in memory Th2 cells even in the absence of IL-4 [11]. In the course of an infection with gut nematodes, signaling via the IL-4Rα has been shown to be critical in the initiation and the development of protective Th2 immunity against primary infection [6,8] and in the development of an optimal memory Th2 response to counter secondary infections [21–24]. However, whether IL-4Rα mediated signaling is required for the maintenance of anti-nematode immunity and the recall of memory responses to secondary nematode infection remains to be determined.

In this study, we show that the temporal impairment of IL-4Rα mediated signaling disrupts already established Th2 immune responses during primary and secondary infections with the model nematode *Nippostrongylus brasiliensis* and limit the host ability to expel the worms. These data suggest an important requirement for sustained IL-4Rα expression to maintain optimal type 2 immune responses and recall of host protective memory responses.

## Results

### Kinetics for the development of type 2 immune responses to *N*. *brasiliensis* in wild-type mice

**Fig 1:**
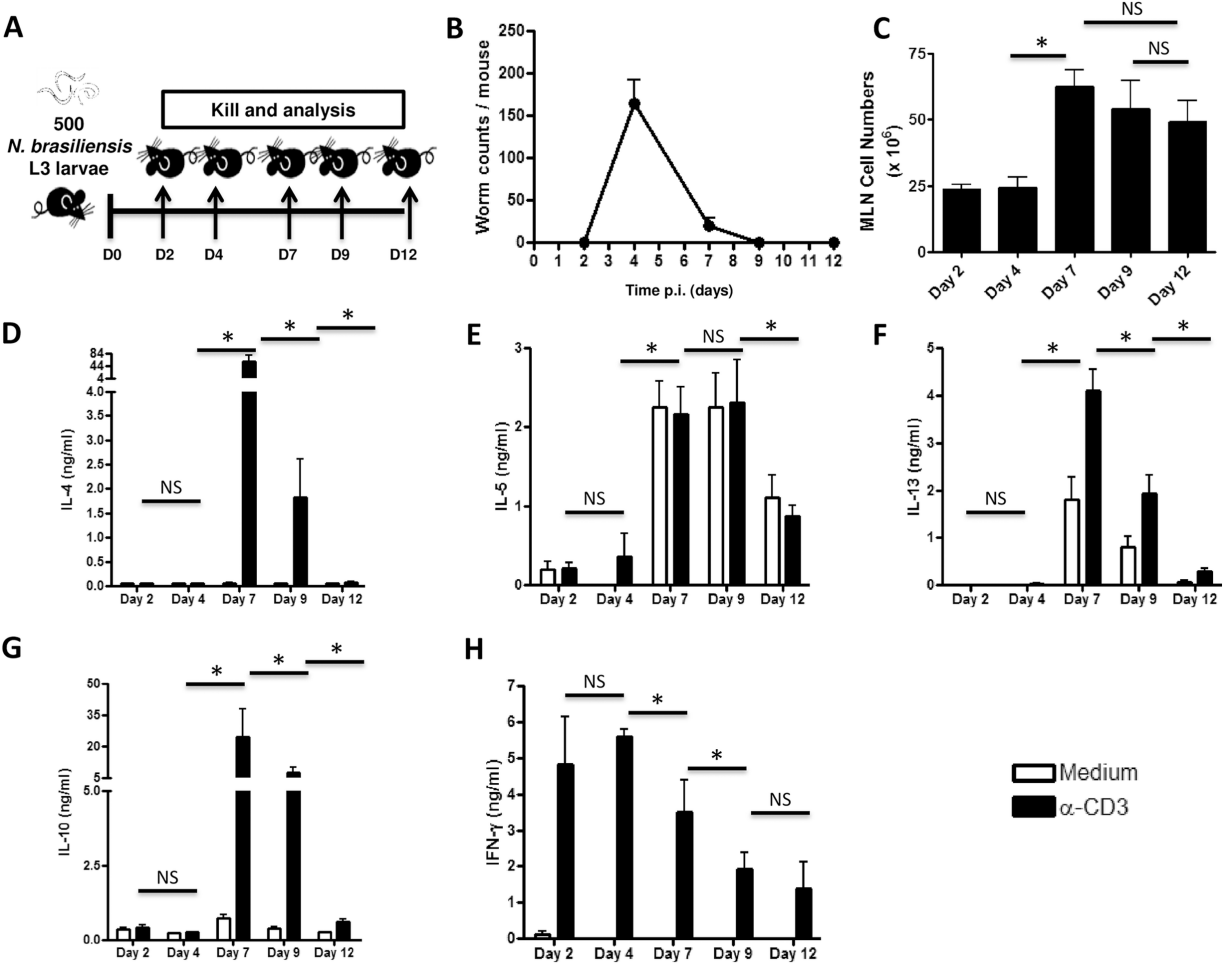
Kinetics of the host response following primary infection with *N*. *brasiliensis*. **A.** Experimental design. **B.** Kinetics of gut worm counts following infection with *N*. *brasiliensis* L3 larvae. **C.** Kinetics of mesenteric lymph node (MLN) total cell counts following infection with *N*. *brasiliensis* L3. (**D-H**). Cytokine release after restimulation of MLN cells isolated at different time points from mice infected with *N*. *brasiliensis* larvae. **D,** IL-4. **E,** IL-5. **F,** IL-13. **G,** IL-10. **H,** IFN-γ. Each experiment was conducted twice with 3–4 mice per group. Data are expressed as mean ± SD. Statistical significance is determined between α-CD3 stimulated values a different time points as per the following legend: NS = p > 0.05; * = p < 0.05; ** = p < 0.01; *** =, p < 0.001; **** = p < 0.0001.

Development of IL-4Rα-driven type 2 immune responses is crucial for the clearance of *N*. *brasiliensis* worms in mice [8]. In order to determine when peak type 2 immune responses develop in response to *N*. *brasiliensis* infection, C57BL/6 mice were infected with 500 L3 *N*. *brasiliensis* larvae and killed at different time points (Fig 1A). The number of worms in the gut peaked at day 4 post-infection and decreased until all the worms were cleared by day 9 post-infection (Fig 1B). Single-cell suspensions of mesenteric lymph nodes (MLN) showed that the number of cells draining into the MLN peaked at day 7 post-infection (Fig 1C). Importantly, stimulation of harvested MLN cells with 20μg/ml α-CD3 revealed that production of Th2 cytokines (IL-4, IL-5, IL-13 and IL- 10) peaked from day 6 (S1 Fig) to day 7 post-infection and diminished at day 9 and 12 post-infection (Fig 1D–1G). Strikingly, α-CD3 stimulation of MLN cells led to the early production of IFN-g peaking between day 2 and day 4 post infection to subsequently drop through day 7, 9 and 12 post infection (Fig 1H) inversely mirroring the kinetics of type 2 cytokines. Collectively, these data demonstrated that type 2 immune responses in the mesenteric lymph nodes peaks at 6 to 7 days post-infection with *N*. *brasiliensis* in C57BL/6 mice.

### Efficient timely interruption of IL-4Rα expression in rosaCreER^T2-/+^IL-4Rα^-/Lox^ mice treated with tamoxifen

**Fig 2:**
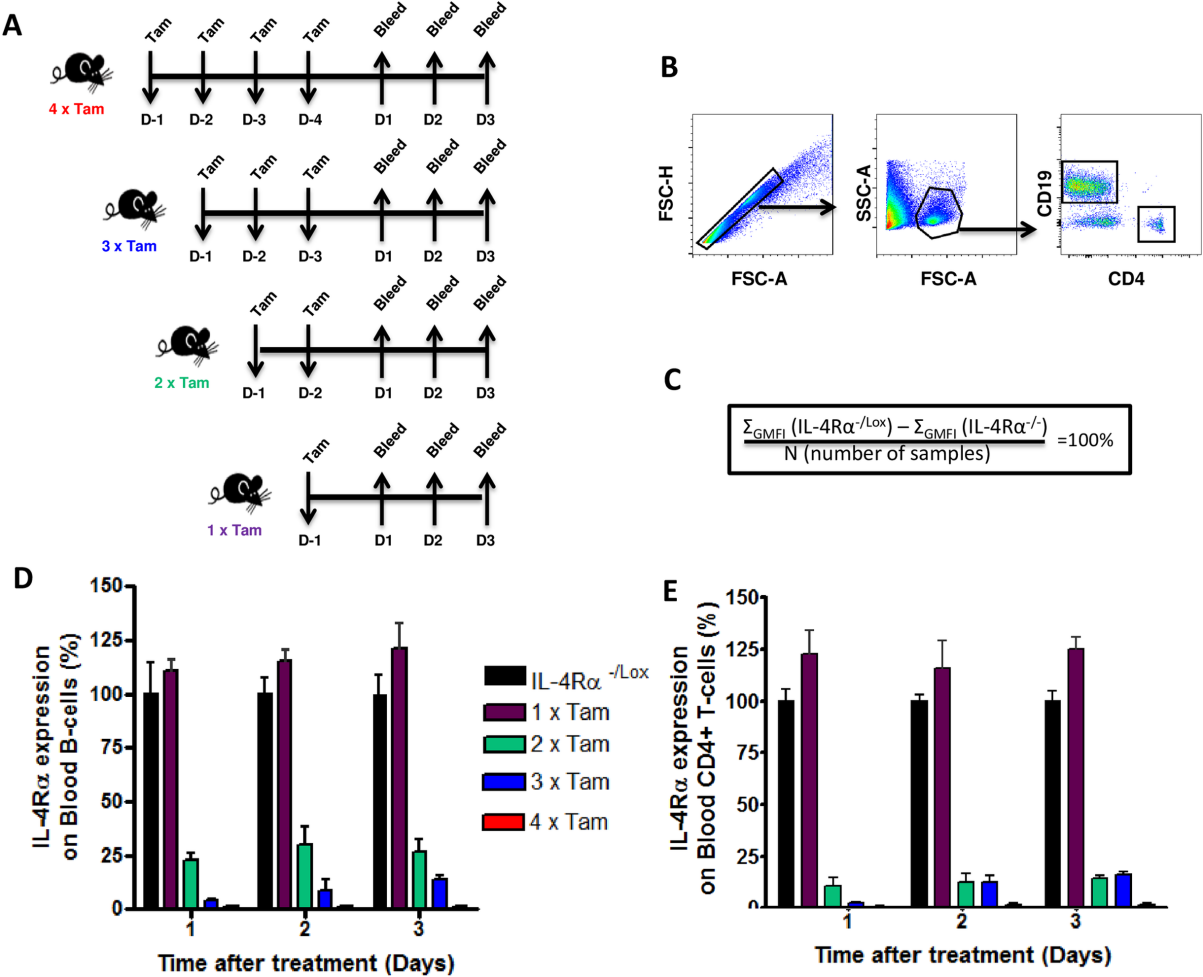
Kinetics of interruption of IL-4Rα expression following tamoxifen administration to i^Cre-/+^IL-4Rα^-/Lox^ mice. **A.** Experimental design. **B.** Gating strategy for Blood CD19^+^CD4^-^ B cells and CD19^-^CD4^+^ T-cells. **C.** Formula for the relative quantification of the levels of IL-4Rα expression on blood B cells. A level of 100% is defined here as the difference between the average IL-4Rα GMFI on cells from control mice (IL-4Rα^-/Lox^) and that of the average IL-4Rα expression on cells from IL-4Rα deficient mice (IL-4Rα^-/-^). The relative expression of IL-4Rα of i^Cre-/+^IL-4Rα^-/Lox^ mice fed with tamoxifen as in **A** is summarized in **D** for Blood CD19^+^ B cells and E for Blood CD4^+^ T-cells. The experiment was conducted with 3–4 mice per group. Data are expressed as mean ± SD.

Our group has recently generated a rosaCreER^T2-/+^IL-4Rα^-/Lox^ (hereafter referred to as i^Cre-/+^IL-4Rα^-/Lox^) mouse model that carries a functional and a floxed IL-4Rα allele that can be timely deleted at any time point by a tamoxifen-responsive *Cre* recombinase (CreER^T2^) upon oral administration of tamoxifen (S2 Fig; Nono *et al*., submitted). Flow cytometry analyses of various tissues and cell types showed the ability of tamoxifen to efficiently (S2A–S2G Fig) and robustly (S2H–S2J Fig) knockdown IL-4Rα expression in the i^Cre-/+^IL-4Rα^-/Lox^ model following tamoxifen administration. Taking advantage of this model, we first sought to determine the timely efficiency of IL-4Rα deletion in i^Cre-/+^IL-4Rα^-/Lox^ mice. Naïve i^Cre-/+^IL-4Rα^-/Lox^ mice were fed tamoxifen by oral gavage for only 1 day before analysis (1× Tam), 2 consecutive days before analysis (2× Tam), 3 consecutive days before analysis (3× Tam) or 4 consecutive days before analysis (4× Tam) (Fig 2A). Blood was collected at day 1, 2 and 3 after each given treatment scheme and IL-4Rα expression on circulating CD19^+^ B cells and CD4^+^ T cells was analyzed by flow cytometry to determine IL-4Rα deletion efficiency (Fig 2A & 2B). IL-4Rα expression was defined as the difference between the average geometric mean fluorescence intensity (GMFI) on the analyzed B cells and the average IL-4Rα GMFI on cells from IL-4Rα deficient mice (Fig 2C). A reference value of 100% was given to the IL-4Rα expression on B cells from IL-4Rα^-/Lox^ mice (Fig 2C–2E). Single treatment with tamoxifen did not interrupt IL-4Rα expression on blood B or T cells of i^Cre-/+^IL-4Rα^-/Lox^ mice as shown by similar levels of receptor expression as IL-4Rα^-/Lox^ littermate control mice until 3 days after the last treatment (Fig 2D & 2E). Treating i^Cre-/+^IL-4Rα^-/Lox^ mice twice with tamoxifen resulted in 75% and 85% reduction of IL-4Rα expression as early as 1 day after the last treatment on circulating CD19^+^ B cells and CD4^+^ T cells respectively, and this reduction in IL-4Rα expression was maintained for up to 3 days following the last treatment (Fig 2D and 2E). i^Cre-/+^IL-4Rα^-/Lox^ mice that were treated for three or four consecutive days displayed almost 100% ablation of IL-4Rα expression in circulating blood B cells (Fig 2D) and blood T cells (Fig 2E) compared to littermate IL-4Rα^-/Lox^ mice. Overall, these data showed that 2 days of treatment with daily doses of tamoxifen are sufficient to efficiently induce considerable IL-4Rα deletion in i^Cre-/+^IL-4Rα^-/Lox^ mice.

### IL-4Rα expression is required for maintenance of protective type 2 immune responses in mice infected with *N*. *brasiliensis*

**Fig 3:**
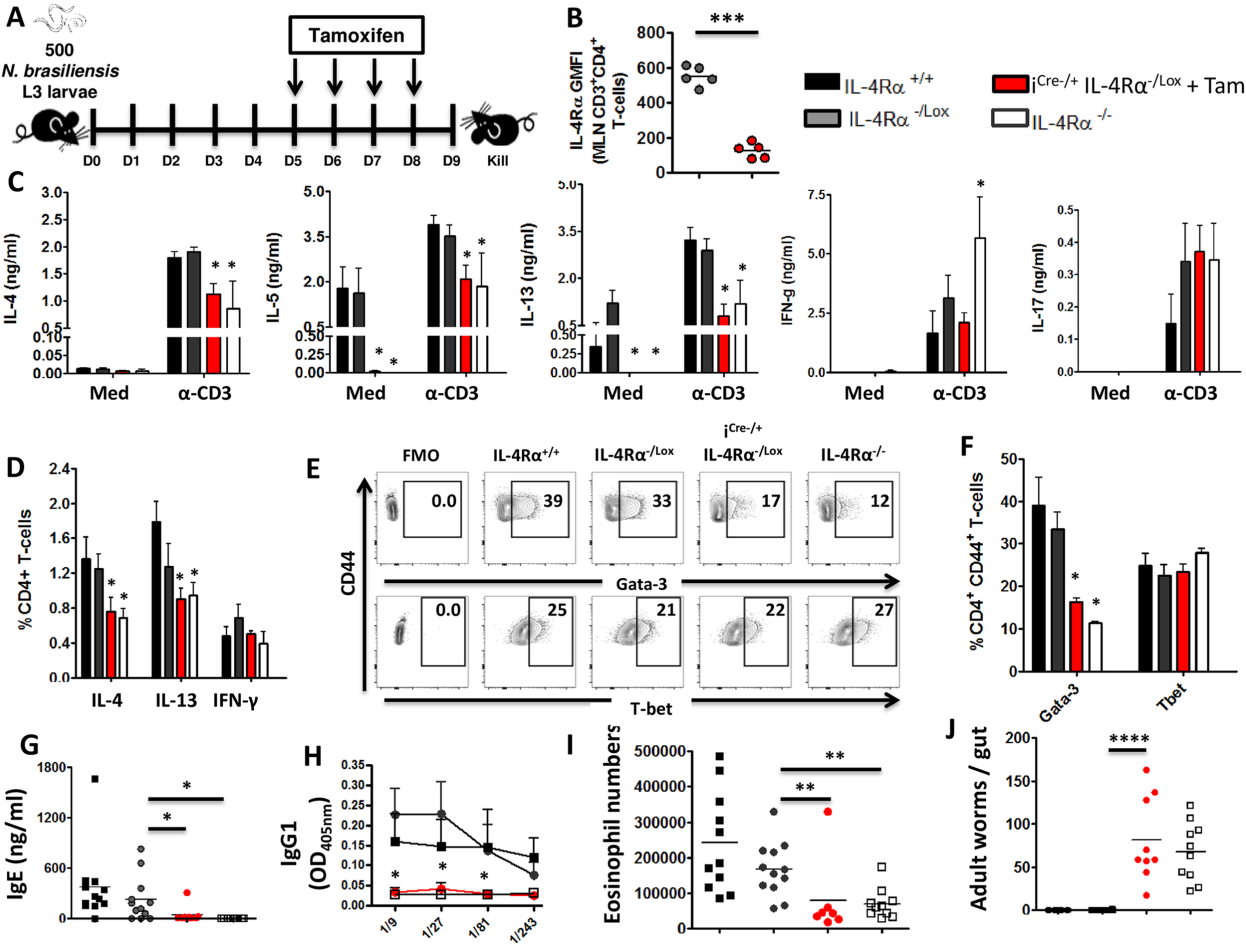
Sustained IL-4Rα mediated signaling is critical for the anti-parasitic type 2 immune responses during primary *N*. *brasiliensis* infection. Mice were infected with 500 L3 *N*. *brasiliensis*, fed with tamoxifen once daily from day 5 to day 8 post infection and killed 9 days post-infection. **A.** Schematic showing the experimental design. **B.** IL-4Rα expression on CD3^+^CD4^+^ T-cells from MLN of *N*. *brasiliensis*-infected mice. **C.** Cytokine production detected by ELISA in the supernatant of restimulated MLN cells. **D.** Percentages of cytokine-producing CD4^+^ T-cells after stimulation with PMA/Ionomycin/Monensin cocktail. **E.** Percentages of transcription factor-expressing CD3^+^CD4^+^ T-cells (*ex-vivo*) summarized in (**F**)**. G.** Total serum IgE in *N*. *brasiliensis-infected* mice. **H.** NbAg-specific serum type 2 antibodies. **I.** Eosinophil (SiglecF^hi^) numbers per MLN. **J.** Worm counts per gut. Each experiment was conducted at least twice with 4–12 mice per group. Data are expressed as mean ± SD; NS = p > 0.05; * = p < 0.05; ** = p < 0.01; *** =, p < 0.001; **** = p < 0.0001.

Although the role of IL-4Rα mediated signaling in initiating and polarizing the development of type 2 immune responses following nematode infections is well established [8,25,26], its requirement in the maintenance of type 2 immune responses is yet to be determined. In order to investigate whether IL-4Rα is required to maintain type 2 immune responses, i^Cre-/+^IL-4Rα^-/Lox^ mice were infected with 500 *N*. *brasiliensis* L3 and IL-4Rα expression was timely impaired between day 6 and day 7 post infection after an optimal Th2 immune responses has already been established as indicated in our kinetics study (S1 Fig), by administering tamoxifen day 5 post infection onwards (Fig 3A). Single cell suspensions were prepared from MLNs and IL-4Rα expression was analyzed by flow cytometry. Timely administration of tamoxifen abrogated IL-4Rα expression on CD3^+^CD4^+^ T cells by almost 3 folds compared to littermate control IL-4Rα^-/Lox^ mice (Fig 3B).

To investigate whether IL-4Rα mediated signaling is required to maintain the production of Th2 cytokines after *N*. *brasiliensis* infection, cytokine responses were determined by ELISA after re-stimulating total MLN cells with 20μg/ml α-CD3 or cells were left un-stimulated (media, Med). Interestingly, timely deletion of IL-4Rα expression after the establishment of peak Th2 immune responses resulted in significantly reduced production of IL-4, IL-5 and IL-13 after α-CD3 stimulation in i^Cre-/+^IL-4Rα^-/Lox^ and IL-4Rα^-/-^ mice compared to both IL-4Rα^+/+^ and IL-4Rα^-/Lox^ control mice (Fig 3C). Production of the canonical type 1 cytokine IFN-γ was not elevated in i^Cre-/+^IL-4Rα^-/Lox^ mice as observed in IL- 4Rα^-/-^ mice, suggesting that timely interruption of IL-4Rα does not result in Th2 to Th1 shift in immune polarization (Fig 3C). Similarly, neither i^Cre-/+^IL-4Rα^-/Lox^ nor IL-4Rα^-/-^ mice had altered IL-17 production after re-stimulation of cells with mitogen (Fig 3C). In agreement with the cytokine profiles, the proportions of MLN CD4^+^ T-cells secreting IL-4 and IL-13 was significantly decreased in mutant mice compared to control mice while the proportions of CD4^+^ T-cells secreting IFN-γ were similar between all strains (S3A and S3D Fig). The canonical innate type 1 cytokine IL-12p70 did not appear to be differentially produced by stimulated MLN cells from IL-4Rα-depleted mice (S3B Fig), further supporting our overall profile of a diminished type 2 and an unaltered type 1 immune response in our inducible model. This was further corroborated by the quantification of effector CD4^+^ T cells expressing the Th2 transcription factor Gata-3, which was reduced by 50% in both i^Cre-/+^IL-4Rα^-/Lox^ and IL-4Rα^-/-^ mice compared to both IL-4Rα^+/+^ and IL-4Rα^-/Lox^ control mice (Fig 3E & 3F). Therefore, these data demonstrated a novel function for IL-4Rα mediated signaling in the maintenance of type 2 immune responses during the course of a nematode infection.

The impact of timely deletion of IL-4Rα expression on humoral immunity was analyzed by determining the levels of antibody titers present in the sera of infected mice by ELISA. Mice treated with tamoxifen displayed reduced titers of type 2 antibody isotypes (total IgE and antigen-specific IgG1 and IgG2a) compared to control mice (Figs 3G, 3H & S3C). Moreover, iCre^-/+^IL-4Rα^-/Lox^ and IL-4Rα^-/-^ mice had significantly reduced number of eosinophils in the MLN compared to control mice (Fig 3I identified as per S3D Fig). Intriguingly, however, we failed to observe a reduction of Arginase-expressing macrophages (S3D, S3E & S3F Fig) in the MLN of our iCre^-/+^IL-4Rα^-/Lox^ mice as opposed to IL-4Rα^-/-^ mice where a significant reduction of these cells was apparent (S3E Fig). These data showed that knocking down IL-4Rα mediated signaling impairs humoral and type 2 innate immune responses in mice infected with *N*. *brasiliensis*.

Finally, we investigated whether the impairment of IL-4Rα expression after the development of an optimal Th2 immune response would impair worm clearance in mice. Interestingly, despite the presence of IL-4Rα early during infection, timely interruption of IL-4Rα mediated signaling after 6 days of infection impaired worm clearance in iCre^-/+^IL-4Rα^-/Lox^ mice similarly to IL-4Rα^-/-^ mice that lacked the receptor from birth (Fig 3J). In contrast, control mice efficiently cleared *N*. *brasiliensis* worms by day 9 post-infection (Fig 3J). This was mechanistically supported by a significantly reduced production of lung (S4A Fig) and gut (S4B Fig) mucins in tamoxifen-fed iCre^-/+^IL-4Rα^-/Lox^ mice. Therefore, these data showed that persistent IL-4Rα mediated signaling is necessary for efficient worm clearance in mice.

### Impaired recall responses in mice deficient of IL-4Rα mediated signaling during secondary *N*. *brasiliensis* infection

**Fig 4:**
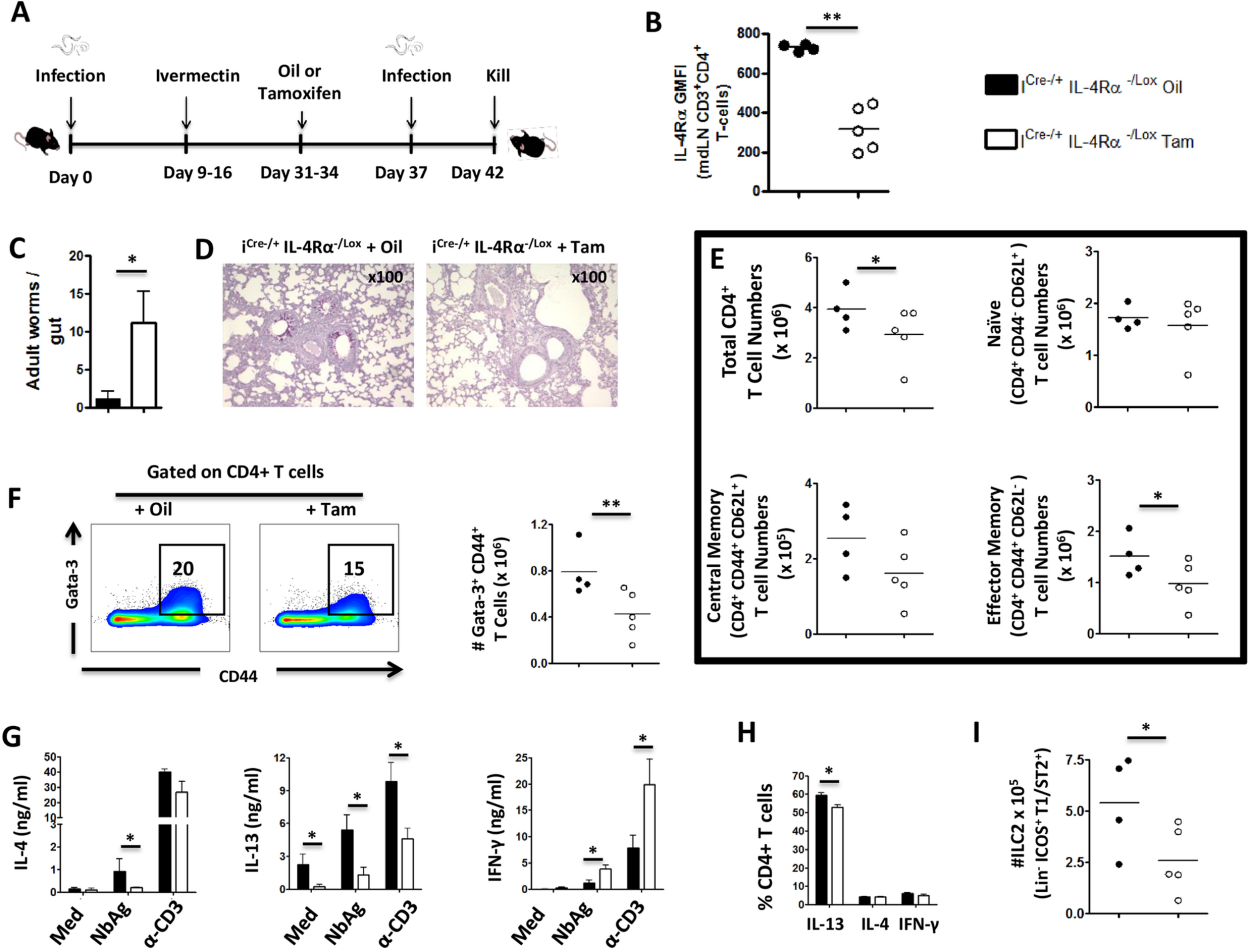
Sustained IL-4Rα mediated signaling is required for recall responses to re-infection with *N*. *brasiliensis*. Mice were infected with 500 L3 *N*. *brasiliensis*, treated with Ivermectin as from day 9 post infection, fed with tamoxifen once daily from day 31 to day 34 then re-infected with 500 L3 *N*. *brasiliensis* at day 37 and killed 5 days post-infection. **A.** Schematic showing the experimental design. **B.** IL-4Rα expression on mdLN CD3^+^CD4^+^ T cells after secondary infection with *N*. *brasiliensis*. **C.** Worm counts per gut. **D.** PAS staining of pulmonary mucus producing goblet cells. **E.** Total numbers of various CD3^+^CD4^+^ T cell subsets (*ex-vivo*). **F.** Percentages and total numbers of Gata-3^+^ CD4^+^ T-cells. **G.** Cytokine release detected by ELISA in the supernatant of mediastinal lymph node cells either left unstimulated primed with the parasite antigen NbAg or restimulated with anti-CD3 antibodies. **H.** Percentages of cytokine-producing mediastinal lymph nodes CD4^+^ T-cells after stimulation with PMA/Ionomycin/Monensin cocktail. **I.** Total ILC2 numbers in mediastinal lymph nodes. Each experiment was conducted at least twice with 4–5 mice per group. Data are expressed as mean ± SD; * = p < 0.05; ** = p < 0.01.

The role of IL-4Rα in recall of host protective type 2 memory responses is unknown due to the lack of appropriate mouse models to study it. Our recently generated inducible IL-4Rα deleting mouse model; i^Cre-/+^IL-4Rα^-/Lox^, allows for timely interruption of IL-4Rα expression after primary *N*. *brasiliensis* infection. Mice (i^Cre-/+^IL-4Rα^-/Lox^) were infected with 500 *N*. *brasiliensis* L3, treated with Ivermectin to allow for complete clearance of the worms and generation of memory responses for 15 days (Fig 4A). i^Cre-/+^IL-4Rα^-/Lox^ mice were then treated with either tamoxifen from day 31 to 34 to induce deletion of IL-4Rα or given vegetable oil as a control (Fig 4A). Mice were re-infected with 500 *N*. *brasiliensis* L3 at day 37 and killed 5 days later (Fig 4A).

Administration of tamoxifen prior to secondary infection successfully reduced the expression of IL-4Rα on mediastinal lymph node (mdLN) CD4^+^ T cells in i^Cre-/+^IL-4Rα^-/Lox^ mice compared to control that were given vegetable oil (Fig 4B). Importantly, timely deletion of IL-4Rα reduced the ability i^Cre-/+^IL-4Rα^-/Lox^ mice of to expel *N*. *brasiliensis* worms compared to control mice given vegetable oil, demonstrating the advantage of an intact IL-4Rα mediated signaling for recall of host protective memory responses (Fig 4C). This was correlated with reduced mucus production in the lungs indicated by the lack of PAS positive cells (Fig 4D). This was further supported by a significant reduction of effector memory CD4^+^ T-cells in the lung-draining mdLN (Figs S5A and 4E). Therefore, these data demonstrated the requirement for IL-4Rα expression in the generation of effector memory cells and the ensuing recall of host memory responses.

Next, we investigated the possible mechanisms responsible for the host reduced ability to recall protective memory responses by analyzing cellular immunity by flow cytometry and ELISA. Interrupting IL-4Rα expression prior to the secondary infection resulted in reduced proportions and total numbers of CD4^+^CD44^+^ T cells expressing Gata-3 compared to control mice given vegetable oil (Fig 4F). Moreover, i^Cre-/+^IL-4Rα^-/Lox^ mice treated with tamoxifen produced significantly less IL-4 and IL-13 after re-stimulation with either *N*. *brasiliensis* antigen (NbAg) or mitogen compared to control mice (Fig 4G). In contrast, mice treated with tamoxifen produced elevated levels of IFN-γ in response to either NbAg or mitogen, suggesting that deleting IL-4Rα resulted in the immune response being skewed towards type 1 responses (Fig 4G). Intracellular flow cytometry revealed a reduced number of IL-13-producing CD4^+^ T cells (Figs S5B and 4H) supporting a reduction of type 2 effector responses. Moreover, mice treated with tamoxifen had significantly reduced number of innate lymphoid cells (ILC2) compared to control mice (Figs S5C and 4I) consistent with the detected expression of this receptor by tissue ILC2 (S5D Fig). Additionally, mediastinal lymph node cells from tamoxifen-fed inducible mice (IL-4Rα knockdown) produced IL-12p70 at a similar level to that of control oil-treated mice (S6A Fig) but secreted significantly more IL-17 following α-CD3 stimulation (S6B Fig) indicating that type-17 responses, similarly to IFN-γ responses (Fig 4G), are increased during secondary *N*. *brasiliensis* infection following IL-4Rα knockdown. Lastly, mice treated with tamoxifen displayed reduced titers of antibody isotypes (total IgE and antigen-specific IgG1 and IgG2a) compared to control mice (S6C, S6D & S6E Fig) indicating a general impairment of the humoral responses. Together, these data showed that IL-4Rα contributes to the recall and maintenance of optimal type 2 immune responses during secondary infection with *N*. *brasiliensis* in mice.

## Discussion

Our studies elucidate previously unknown functions of IL-4Rα mediated signaling beyond its role in the establishment of Th2 immune responses during *N*. *brasiliensis* infection. By disrupting the host ability to maintain pre-established Th2 immune responses during primary *N*. *brasiliensis* infection and clear the infection, the interruption of IL-4Rα mediated signaling reveals a unique role of this signaling axis in the maintenance of anti-nematode Th2 immune responses. Moreover, the abrogation of IL-4Rα mediated signaling prior to secondary *N*. *brasiliensis* infection led to a diminished recall of Th2 responses and the reduced ability of the host to rapidly clear the infection, indicating a role for this signaling axis in the recall response to secondary *N*. *brasiliensis* infection.

Our understanding of the contribution of IL-4Rα mediated signaling during protective immune responses against nematodes has gradually increased over the last years. Seminal studies with rodent models of nematode infections revealed early on a critical role for Th2 immune responses in the host protection against parasitic gastrointestinal nematodes [3]. Owing to its critical role in the establishment of Th2 responses [27], a pivotal role was then uncovered for IL-4Rα mediated signaling in the host protection against parasitic nematodes [8]. Using IL-4Rα deficient mice, several studies further demonstrated the necessity for IL-4Rα mediated signaling in the development of anti-nematode Th2 immune responses and the generation of Th2 memory responses [4,6,19]. An exciting yet unanswered question was how the maintenance of such Th2 responses, which are paramount to guarantee sterilizing anti-nematode immunity, is achieved. Particularly, it was still not clear whether IL-4Rα mediated signaling was required for the maintenance of established (already developed) Th2 immune responses and the recall response of memory Th2 immune responses during nematode infections. To address these questions, we utilized a recently established inducible IL-4Rα deficient mouse model (i^Cre-/+^IL-4Rα^-/Lox^), where IL-4Rα deletion was induced by feeding mice tamoxifen, an oestrogen analogue (Nono *et al*, submitted). This model enabled the knockdown of IL-4Rα after the development of optimal Th2 responses in the presence of the receptor following primary *N*. *brasiliensis* infection.

As adult worms develop in the small intestines during infection of mice with *N*. *brasiliensis*, a strong host protective Th2 immune response is evoked to facilitate worm expulsion [2,5,8]. In our study, we showed that C57BL/6 mice clear the infection approximately 9 days post infection. This is consistent with the current consensus in the literature that immunocompetent mice clear *N*. *brasiliensis* primary infection at 9 days post infection [28]. Production of Th2 cytokines (IL-4, IL-5, IL-10 and IL-13) by MLN cells similarly peaked at day 6–7 post infection and gradually declined thereafter. As per the literature [25], IL-4Rα mediated signaling is dispensable for the initial development of such Th2 cells in response to *N*. *brasiliensis* antigens but the subsequent expansion of these Th2 cells in a competitive environment that also contains Th1 potential is positively influenced by IL-4Rα mediated signaling [25]. Primary infection in IL-4Rα^-/-^ mice with *N*. *brasiliensis* confirmed the crucial role of IL-4Rα in the establishment of optimal Th2 immunity, as IL-4Rα^-/-^ mice displayed a poor ability to expel worms from their gut. This was associated with reduced CD4^+^ T cell specific expression of Gata-3, abrogated IL-13 production and reduced eosinophil expansion in the mesenteric lymph nodes, all known to mediate worm expulsion during *N*. *brasiliensis* infection [2,5,6]. Blocking IL-4Rα mediated signaling in infected mice after the establishment of peak Th2 immune responses in our newly generated mouse model of temporal inducible deletion of IL-4Rα (Nono *et al*. submitted), interfered with the host expulsion of the *N*. *brasiliensis* worms and reduced the magnitude of the established Th2 immune response as judged by reduced IL-4^+^ T-cells, Gata-3^+^ T-cells and eosinophils in the gut-draining MLN and that of mucus-producing goblet cells in the gut. Complementarily, serum analyses showed reduced titers of all tested antibody isotypes, indicating a general disruption of humoral immunity once IL-4Rα mediated signaling is impaired. Overall, this is consistent with our observations on a well established chronic Th2 model of infection with *Schistosoma mansoni* where the knockdown of IL-4Rα using our newly defined inducible mouse model led to a significant drop in type 2 responses (Nono *et al*, submitted). Taken together, our data presents a strong case for a necessity of sustained IL-4Rα mediated signaling to maintain the stability of established type 2 immune responses during helminth infections.

Interrupting IL-4Rα mediated signaling prior to secondary infection in inducible IL-4Rα deficient mice diminished the recall of protective memory response. Interestingly, however, IL-4Rα knockdown did not prevent the recall of a strong type 2 response as judged by IL-4 levels and the considerable reduction of the number of remnant worms in the gut of IL-4Rα knockdown mice during secondary infections. This argues for either a limited role of IL-4Rα signaling in the recall of protective responses against gut nematodes or could also simply reflects the host need of a minimal level of intact IL-4Rα signaling to elicit protection against nematodes. Further validation with a model of inducible knockout rather than knockdown of IL-4Rα would shed more light on what the situation really is i.e. whether a minimal level of intact IL-4Rα signaling is sufficient to drive host protective response to secondary Nematode infection or whether hitherto unappreciated IL-4Rα-independent pathways might drive such protective Th2 memory and responses. As of yet, our findings of remnant gut worms following IL-4Rα knockdown prior to secondary infection strongly suggests that IL-4Rα mediated signaling also contributes to build a more efficient protective immunity against *N*. *brasiliensis*. This conclusion adds to previous findings that have made use of IL-4Rα^-/-^ mice where IL-4Rα is already absent during the primary infection [8,18,24]. In those studies, whether IL-4Rα absence negatively affected the development of memory Th2 cells or simply impaired the subsequent recall response could not be dissociated. Our present findings, using our model of inducible IL-4Rα deletion where IL-4Rα is only removed from infected mice prior to secondary infection argues for a contributing role of IL-4Rα in the memory Th2 response during secondary *N*. *brasiliensis* infection. However, this does not exclude a possible role of IL-4Rα in the development of memory Th2 during primary *N*. *brasiliensis* infections. The use of a transient model of interference with IL-4Rα mediated signaling uniquely during the priming phase of our model of secondary *N*. *brasiliensis* infection should provide complementary information in this respect. As of now, the observed reduction of protective memory Th2 responses after IL-4Rα removal prior to secondary *N*. *brasiliensis* infection was accompanied by reduced production of Th2 cytokines by mediastinal lymph node cells, and CD4^+^ T-cells in particular. Given that IL-4Rα mediated signaling is most likely critical for the positive feedback loop driving memory type 2 immune responses [21] that mediate the host protective immunity against re-infection with *N*. *brasiliensis* [21], our findings of a contributing role for a sustained IL-4Rα mediated signaling on the recall immunity to *N*. *brasiliensis* strongly stand to reason.

IL-4Rα is required for the development of *N*. *brasiliensis*-specific Th2 memory cells [24]. Less well understood is the role of this factor in the maintenance and recall of established memory Th2 cells. In our study, the absolute number of CD4^+^ T cells in the mediastinal lymph nodes draining the lung, a site of protective immunity to recall response to secondary *N*. *brasiliensis* infection [5], was reduced in infected and tamoxifen-fed i^Cre-/+^IL-4Rα^-/Lox^ mice compared to control i^Cre-/+^IL-4Rα^-/Lox^ mice given vegetable oil. These data imply that IL-4Rα is important for the expansion of CD4^+^ T cells in the mediastinal lymph nodes during secondary *N*. *brasiliensis* infection. Moreover, the impairment of IL-4Rα mediated signaling during secondary *N*. *brasiliensis* infection considerably diminished the number of effector memory CD4^+^ T cell populations while naïve CD4^+^ T cell numbers were not affected. Interestingly, naïve i^Cre-/+^IL-4Rα^-/Lox^ mice did not have reduced number of CD4^+^ T cells in lymphoid tissue when compared to wild type mice indicating that i^Cre-/+^IL-4Rα^-/Lox^ mice are phenotypically similar to wild type mice at baseline. These findings altogether suggest that IL-4Rα is required for the expansion of memory Th2 cells during *N*. *brasiliensis* infection. Furthermore, the expression of Gata3, a master transcription factor in Th2 development and stabilizer of Th2 commitment [11] was significantly reduced in effector CD4^+^ T cells following IL-4Rα removal prior to secondary *N*. *brasiliensis* infection. This was paralleled by a reduced production of Th2 cytokines and an increase production of the Th1 cytokine IFN-γ by mediastinal lymph node cells. These findings are consistent with the triple role of Gata3 in the promotion of Th2 immune responses, the expansion of Th2 cells and the inhibition Th1 cell-specific factors [29]. It can therefore be concluded that IL-4Rα removal during recall response to secondary *N*. *brasiliensis* infection reduced Gata-3 expression and consequently diminished Th2 responses and the inhibition of opposing Th1 responses. B cell-driven antibody responses were significantly impaired as judged by reduced titers of both type 1 and type 2 antibody isotypes during recall response to secondary *N*. *brasiliensis* infection following IL-4Rα removal, further indicating a general disruption of humoral immunity.

In our study, IL-4Rα removal during primary and secondary *N*. *brasiliensis* infections led to a drastic reduction of mucus production in the gut (primary infection) and lungs (secondary infection). Our findings are consistent with previous studies that reported a reduced goblet cell metaplasia [30] and a diminished mucus production in the epithelial lining of small bronchi in the lung sections [31] of IL-4Rα–deficient deficient mice following challenge with allergens when compared to IL-4Rα-responsive controls. This further reinforces the critical role of sustained IL-4Rα mediated signaling for the host response to *N*. *brasiliensis* infections as mucins are an important innate components of host immunity [32]. In fact, mucins are gel-forming glycoproteins produced by goblet and paneth cells in the gut [32] and Clara cells of the lung airways [33] that ensure mucosal protection against pathogens like *N*. *brasiliensis* through the physical barrier they constitute, but also via other proteins in the secretions like the antimicrobial peptide Relmβ (FIZZ2) which may impair worm fitness [34]. Furthermore, our study reveals that group 2 innate lymphoid cells, a major source of mucin-inducing IL-13 were significantly reduced in mediastinal lymph nodes following IL-4Rα removal during secondary *N*. *brasiliensis* infection. This further aligns with the reported impairment of Th2 responses following IL-4Rα removal during *N*. *brasiliensis* infection, given the established mutual support system between ILC2 and Th2 cells during *N*. *brasiliensis* infections [17]. Thus, IL-4Rα removal even after establishment of type 2 responses compromised the maintenance of these responses during primary as well as secondary *N*. *brasiliensis* infections.

In conclusion, our study reveals novel facets of the role of IL-4Rα during type 2 immune responses whereby IL-4Rα mediated signaling appears to contribute not only to the initiation and expansion but also to the maintenance and recall of pre-established host protective Th2 responses during *N*. *brasiliensis* infections.

## Material and methods

### Larvae, animals and ethics statement

IL-4Rα^-/-^, IL-4Rα^-/Lox^, CreER^T2^ and IL-4Rα deleting mouse strain (i^Cre-/+^IL-4Rα^-/Lox^) mice on a C57/BL6 background were previously described (Nono *et al*., submitted). Mice were maintained in the University of Cape Town specific pathogen-free animal facility in accordance with the guidelines established by the institutional Animal Research Ethics committee. *N*. *brasiliensis* larvae were kindly provided by Klaus Erb (Wurzburg, Germany) and institutionally approved to be used in animal infection studies under strict recommendation of the South African national guidelines and of the University of Cape Town practice for laboratory animal procedures as outlined in protocols 012/054, 014/021, 016/024 and 017/002 approved by the Animals Research Ethics Committee of the University of Cape Town. Both male and female mice aged 6–12 weeks were used for all experiments. Care was taken to minimize animal suffering.

### Tamoxifen administration

To activate *il-4rα* gene excision by CreER^T2^, Tamoxifen (Sigma, Deisenhofen, Germany) solubilized in vegetable oil was administered by oral gavage to mice for four consecutive days (2.5mg/day).

### *N*. *brasiliensis* infection

#### Primary infection

Mice were injected subcutaneously with 500 *N*. *brasiliensis* L3 suspended in 0.65–0.9% NaCl using 21-G needle (Braun, Melsungen, Germany). Mice were killed 9 days post-infection, tissue samples were collected for analyses and adult worms in the gut were enumerated.

#### Secondary infection

Mice were initially injected with 500 *N*. *brasiliensis* L3, orally treated with 10 mg/ml Ivermectin in drinking water at nine days post-infection and shelved for 21 days prior to a secondary subcutaneous infection with 500 *N*. *brasiliensis* L3. Mice were killed 5 days post secondary infection by halothane inhalation and exsanguination.

### *N*. *brasiliensis* intestinal worm counts

Small intestines were removed from infected mice, the lumen was exposed by dissection and suspended in 0.65% NaCl. The intestines were incubated at 37°C for 4 h to allow for migration of the worms out of the lumen after which they were enumerated under a dissecting microscope (Nikon Eclipse).

### Flow cytometry

Il-4Rα surface expression was detected on lymph node cells by phycoerythrin (PE) anti-CD124 (IL-4Rα, M-1). Cell subpopulations were identified with Alexa Fluor 700, BD Horizon V500, BD Horizon V450, PerCP-Cy5.5, APC, APC-Cy7, Fluoroscein isothiocyanate, PE, PE-Cy7 or biotinylated monoclonal antibodies against CD3, CD4, CD19, Lineage, Gata-3, IL-4, IL-13, IFN-γ, IL-10, SiglecF, T1/ST2, ICOS. Biotin-labeled antibodies were detected by Allophycocyanin or Percpcy5.5. For staining, cells (1x 10^6^) were labeled and washed in PBS, 3%FCS and 0.1% NaN3. Between each step of staining, cells were washed extensively. For intracellular cytokine staining, cells were restimulated with a cocktail of PMA/Ionomycin/Monensin for 4–12 h at 37°C then fixed in 2% PFA, permeabilized and cytokine production was analyzed as previously described [28]. For intranuclear staining, a commercially available transcription buffer set (BD Bioscience) was used as per the manufacturer’s instructions. All antibodies were from BD Pharmingen (San Diego, CA) except where noted otherwise. Stained cells were then acquired on a LSR Fortessa machine (BD Immunocytometry system, San Jose, CA, USA) and data were analyzed using Flowjo software (Treestar, Ashland, OR, Usa).

### *Ex vivo* restimulation

Single cell suspensions from lymph node cells of *N*. *brasiliensis* -infected animals were prepared by pressing the MLN through 70 μm cell-strainers. Cells were resuspended in complete IMDM (Gibco) supplemented with 10% FCS (Gibco) and Penicillin and Streptomycin (100 U/ml and 100 μg/ml, Gibco). The cells were cultured at 1×10^6^ cells/ml in 96-well plates coated with α-CD3 (20 μg/ml) or supplemented with NbAg (20 μg/ml) and incubated at 37°C in a humidified atmosphere containing 5% CO_2_. Supernatants were collected after 72 h and cytokines were measured by sandwich ELISA as previously described [28].

### Serum antibody titers

*N*. *brasiliensis* antigen-specific serum antibody isotypes and total IgE titers from infected mice were determined as follows. Blood was collected in serum separator tubes (BD Bioscience, San Diego, CA) and centrifuged at 8 000×g for 10 min at 4°C to separate serum. The flat-bottom 96-well plates were coated with 10 μg/ml NbAg, blocked with 2% (w/v) milk powder for 2 h at 37°C and samples were loaded and incubated overnight at 4°C. Alkaline phosphatase labeled secondary antibody was added and incubated for 2 h at 37°C. The plates were developed by addition of 4-nitrophenyl substrate (Sigma). The absorbance was read at 405 nm using VersaMax microplate spectrophotometer (Molecular Devices, Germany).

### Histology

Tissue sample were fixed in buffered 4% (v/v) formaldehyde, embedded in paraffin wax and cut into 5 μm sections. The sections were stained with periodic acid-Schiff reagent (PAS) in order to visualize mucus producing goblet cells. The sections were analyzed under a light microscope.

### Statistics

Statistical analysis was conducted using GraphPad Prism 4 software (http://www.prism-software.com). Data were calculated as mean ± SD. Statistical significance was determined using the unpaired Student's t test, One-Way or Two-Way ANOVA with Bonferroni's post test, defining differences to C57BL/6, IL-4Rα^-/Lox^ or oil-treated i^Cre-/+^IL-4Rα^-/Lox^ as significant (*, p≤0.05; **, p≤0.01; ***, p≤0.001).

## Supporting information

S1 Fig Refined Th2 kinetics in MLN following primary *N*. *brasiliensis* infection. Mice were infected with 500 L3 *N*. *brasiliensis*, and killed 4,6, 9 and 12 days post-infection. MLN cells were cultured unstimulated or stimulated with α-CD3 for 72 hours before measurement IL-4 in culture supernatants by ELISA. Data represents 4 mice per group. Data are expressed as mean ± SD; NS = p > 0.05; * = p < 0.05; ** = p < 0.01; *** =, p < 0.001; **** = p < 0.0001.
(TIF)

S2 Fig Characterization of the rosaCre^-/+^ IL-4Rα^-/Lox^ deleting mouse model (iCre^-/+^ IL-4Rα^-/Lox^ mice). **A.** Experimental set-up. **B.** Gating strategy for F4/80^+^ CD11b^+^ macrophages. **C.** IL-4Rα GMFI in peritoneal macrophages. **D.** IL-4Rα GMFI on Lung macrophages. **E.** Gating strategy for Ly6G^+^ CD11b^+^ Neutrophils. **F.** IL-4Rα GMFI on spleen neutrophils. **G.** IL-4Rα GMFI on lung neutrophils. **H.** Experimental set-up to assess the stability of IL-4Rα deletion on blood B cells over time following Tamoxifen administration to inducible iCre^-/+^ IL-4Rα^-/Lox^ mice. **I.** Gating strategy for Blood B cells. **J.** IL-4Rα relative GMFI on Blood B cells (100% for **IL-4Rα**^**-/Lox**^ and 0% for **IL-4Rα**^**-/-**^) over time following Tamoxifen administration to inducible iCre^-/+^ IL-4Rα^-/Lox^ mice.
(TIF)

S3 Fig Representative gating strategies, cytokine production and serum antibody levels during primary Nb infection. **A.** Cytokine-producing MLN CD4^+^ T-cells. **B.** IL-12p70 produced by MLN cells. **C.** Serum levels of Nb-antigen specific IgG2a. **D.** MLN eosinophils and Arginase+ F4/80^+^ CD11b^+^ Macrophages. **E.** Total numbers of MLN Arg-1^+^ F4/80^+^ Macrophages. **F.** Arg-1 GMFI in MLN macrophages. Each experiment was conducted at least twice with 4–12 mice per group. Data are expressed as mean ± SD; NS = p > 0.05; * = p < 0.05; ** = p < 0.01; *** =, p < 0.001; **** = p < 0.0001.
(TIF)

S4 Fig Sustained IL-4Rα mediated signaling is critical for the production of lung and gut mucins during primary *N*. *brasiliensis* infection. Mice were infected with 500 L3 *N*. *brasiliensis*, fed with tamoxifen once daily from day 5 to day 8 post infection and killed 9 days post-infection. **A.** PAS staining of pulmonary mucus producing goblet cells from *N*. *brasiliensis* infected mice. **B.** PAS staining of mucus producing goblet cells in the intestinal tissue. Data are representative of two independent experiments.
(TIF)

S5 Fig Representative gating strategies and scatter plots in mediastinal lymph nodes during secondary Nb infection. **A.** Gating strategy for MLN CD4^+^ T cell sub-populations. **B.** Cytokine-producing CD4^+^ T cells. **C.** ILC2. **D.** IL-4Rα GMFI in mdLN ILC2.
(TIF)

S6 Fig Cytokines and antibodies during secondary Nb infection. **A.** IL-12p70 from the supernatant of mdLN cells. **B.** IL-17 from the supernatant of mdLN cells. **C.** Serum IgE levels. **D.** Serum levels of Nb Antigen-specific IgG1 levels. **E.** Serum levels of Nb Antigen-specific IgG2a levels.
(TIF)
