# Supplementary figures and images for: Interleukin-4 receptor alpha is still required after Th2 polarization for the maintenance and the recall of protective immunity to Nematode infection
Source: PLoS Negl Trop Dis. 2017 Jun 26;11(6):e0005675. doi: 10.1371/journal.pntd.0005675 (PMC5501681; doi:10.1371/journal.pntd.0005675)

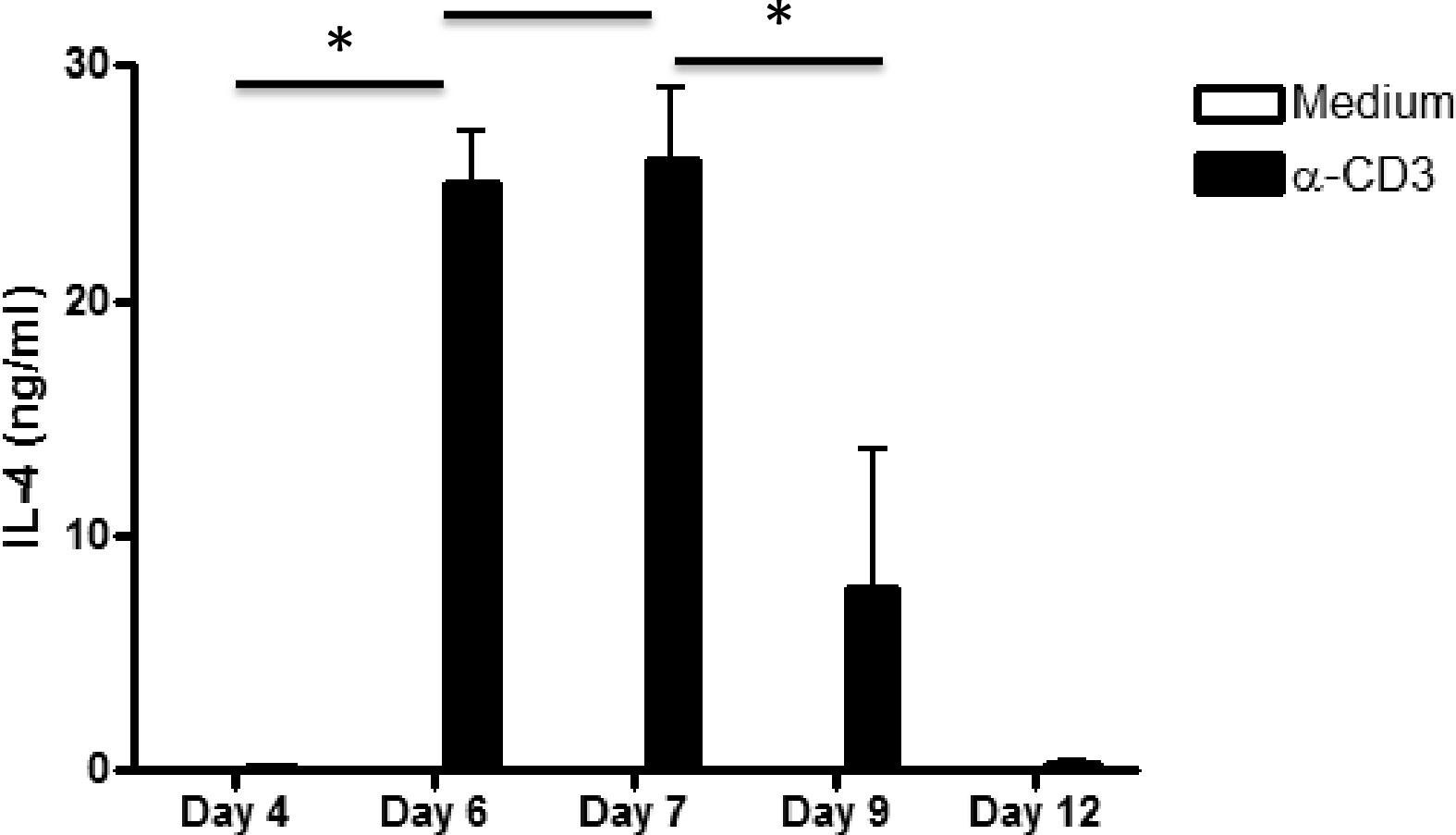

Supplement: S1 Fig — Mice were infected with 500 L3 N. brasiliensis, and killed 4,6, 9 and 12 days post-infection. MLN cells were cultured unstimulated or stimulated with α-CD3 for 72 hours before measurement IL-4 in culture supernatants by ELISA. Data represents 4 mice per group. Data are expressed as mean ± SD; NS = p > 0.05; * = p < 0.05; ** = p < 0.01; *** =, p < 0.001; **** = p < 0.0001. (TIF) [file pntd.0005675.s001.tif]

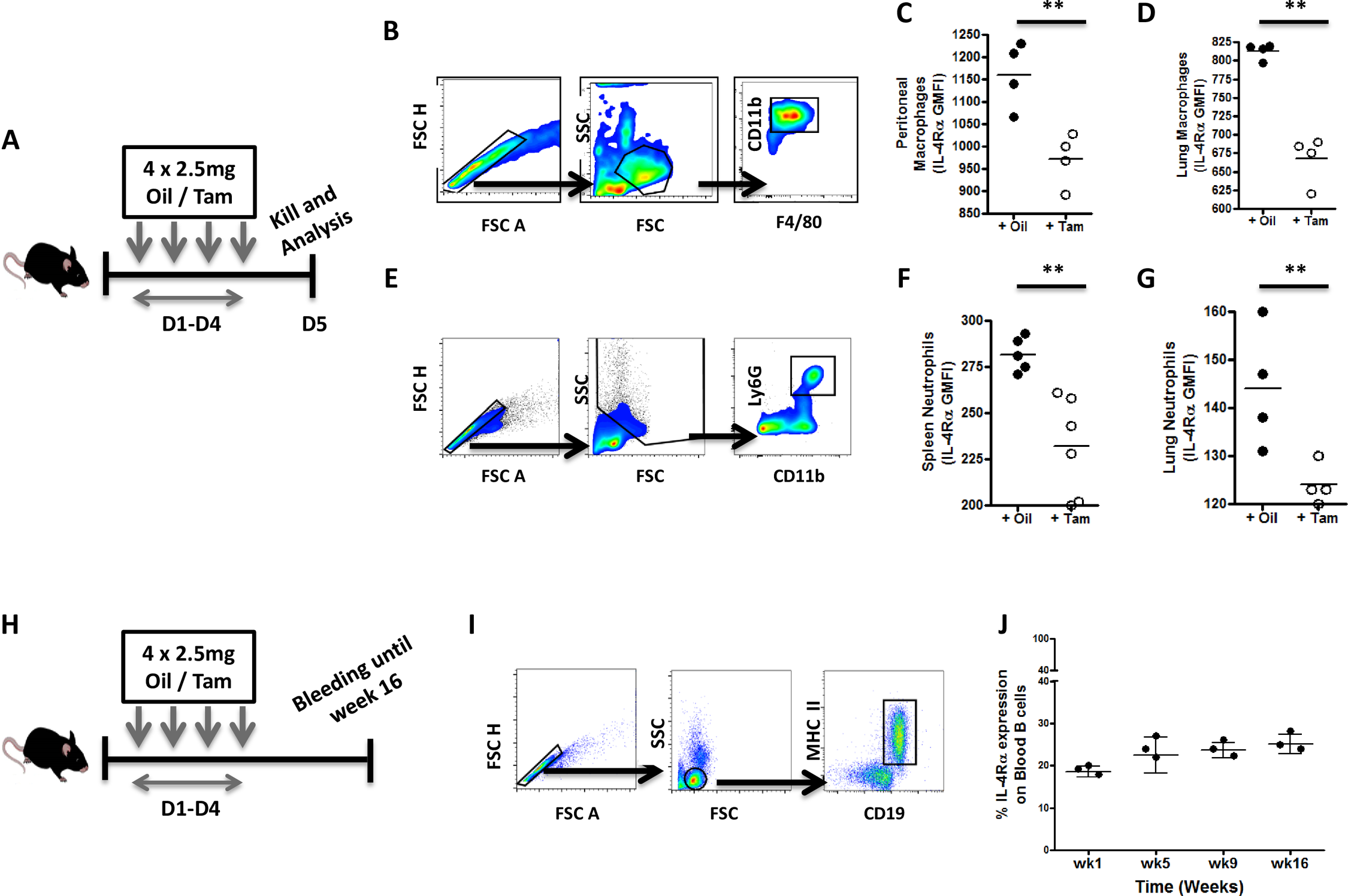

Supplement: S2 Fig — A. Experimental set-up. B. Gating strategy for F4/80+ CD11b+ macrophages. C. IL-4Rα GMFI in peritoneal macrophages. D. IL-4Rα GMFI on Lung macrophages. E. Gating strategy for Ly6G+ CD11b+ Neutrophils. F. IL-4Rα GMFI on spleen neutrophils. G. IL-4Rα GMFI on lung neutrophils. H. Experimental set-up to assess the stability of IL-4Rα deletion on blood B cells over time following Tamoxifen administration to inducible iCre-/+ IL-4Rα-/Lox mice. I. Gating strategy for Blood B cells. J. IL-4Rα relative GMFI on Blood B cells (100% for IL-4Rα-/Lox and 0% for IL-4Rα-/-) over time following Tamoxifen administration to inducible iCre-/+ IL-4Rα-/Lox mice. (TIF) [file pntd.0005675.s002.tif]

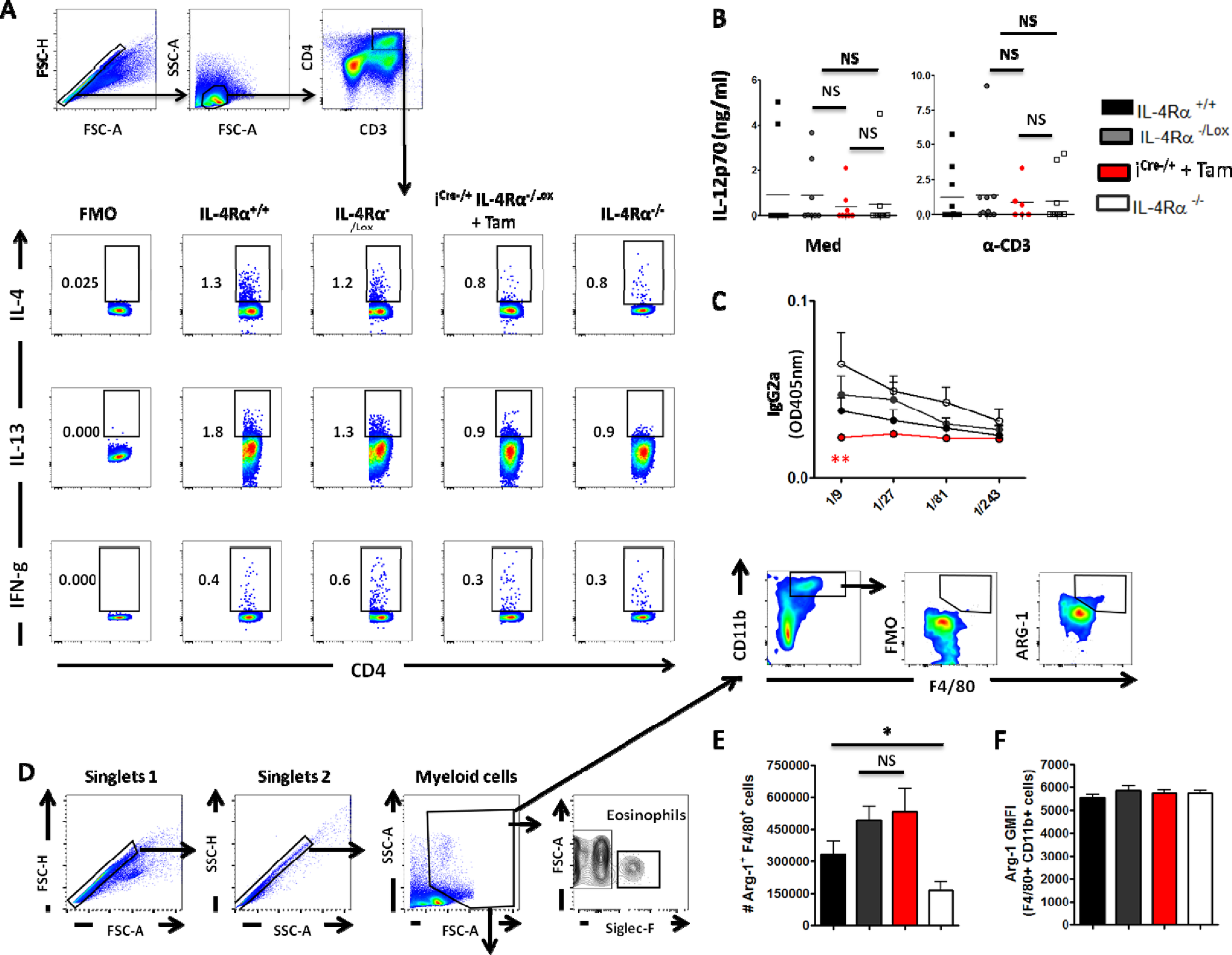

Supplement: S3 Fig — A. Cytokine-producing MLN CD4+ T-cells. B. IL-12p70 produced by MLN cells. C. Serum levels of Nb-antigen specific IgG2a. D. MLN eosinophils and Arginase+ F4/80+ CD11b+ Macrophages. E. Total numbers of MLN Arg-1+ F4/80+ Macrophages. F. Arg-1 GMFI in MLN macrophages. Each experiment was conducted at least twice with 4–12 mice per group. Data are expressed as mean ± SD; NS = p > 0.05; * = p < 0.05; ** = p < 0.01; *** =, p < 0.001; **** = p < 0.0001. (TIF) [file pntd.0005675.s003.tif]

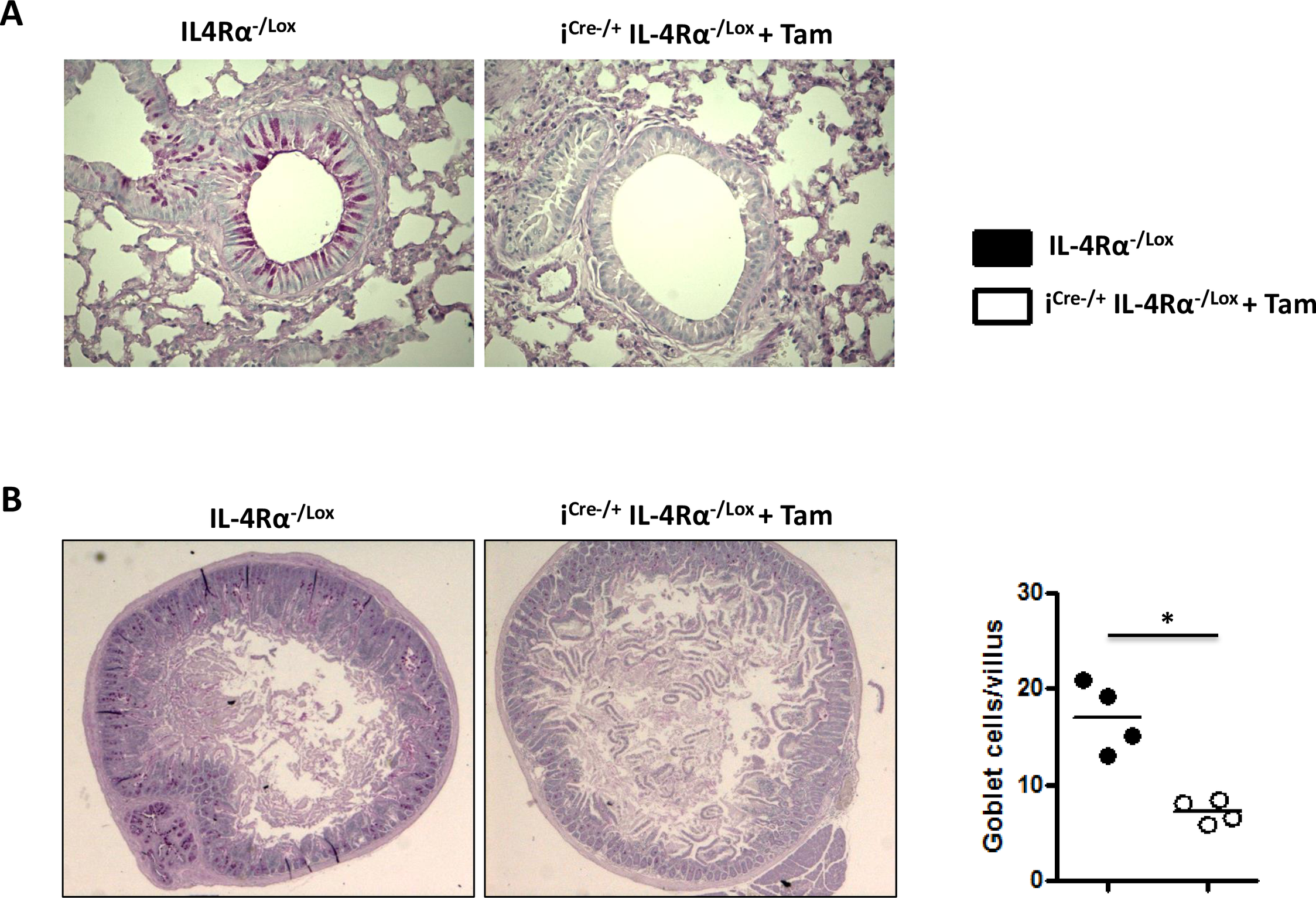

Supplement: S4 Fig — Mice were infected with 500 L3 N. brasiliensis, fed with tamoxifen once daily from day 5 to day 8 post infection and killed 9 days post-infection. A. PAS staining of pulmonary mucus producing goblet cells from N. brasiliensis infected mice. B. PAS staining of mucus producing goblet cells in the intestinal tissue. Data are representative of two independent experiments. (TIF) [file pntd.0005675.s004.tif]

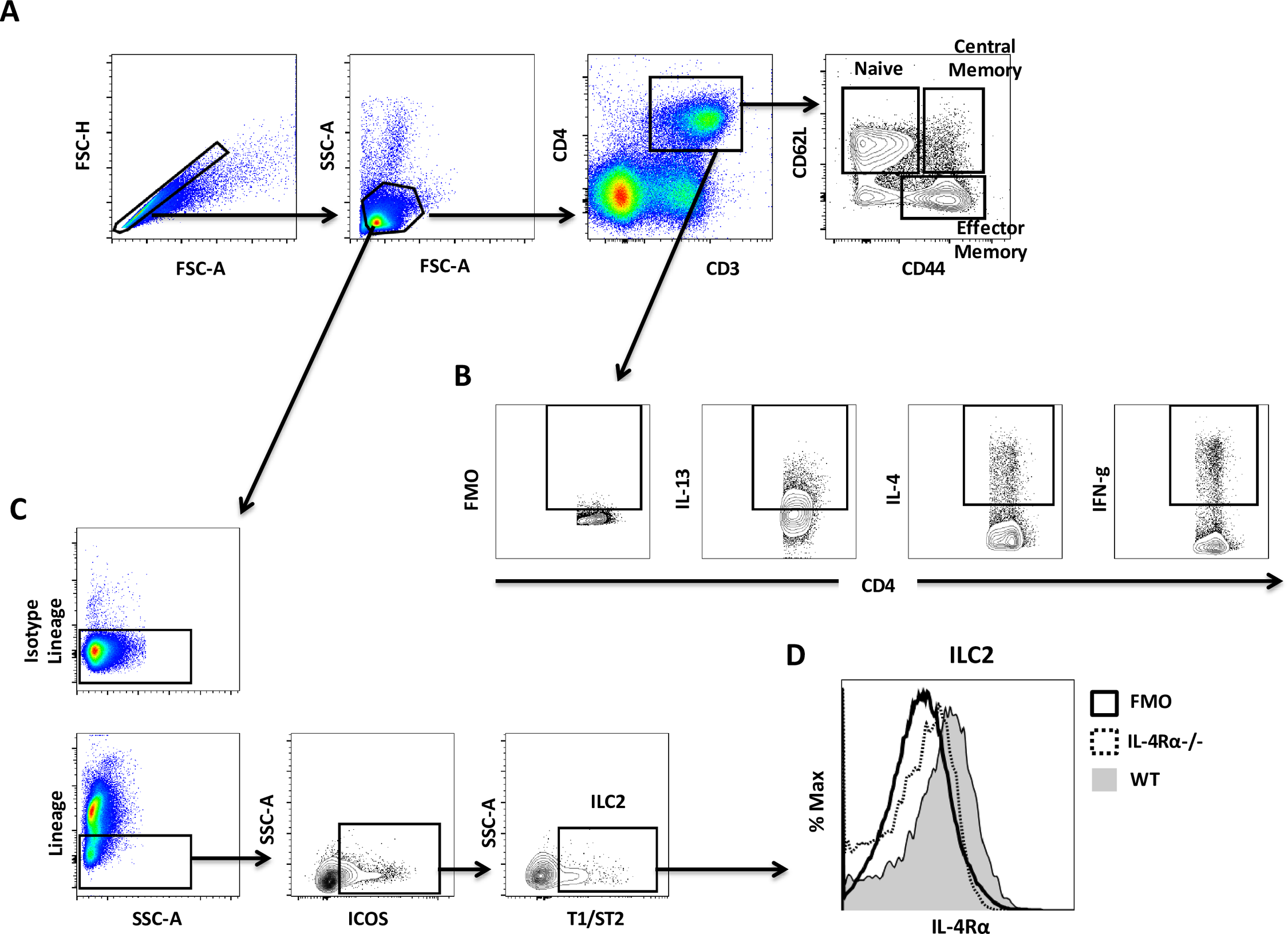

Supplement: S5 Fig — A. Gating strategy for MLN CD4+ T cell sub-populations. B. Cytokine-producing CD4+ T cells. C. ILC2. D. IL-4Rα GMFI in mdLN ILC2. (TIF) [file pntd.0005675.s005.tif]

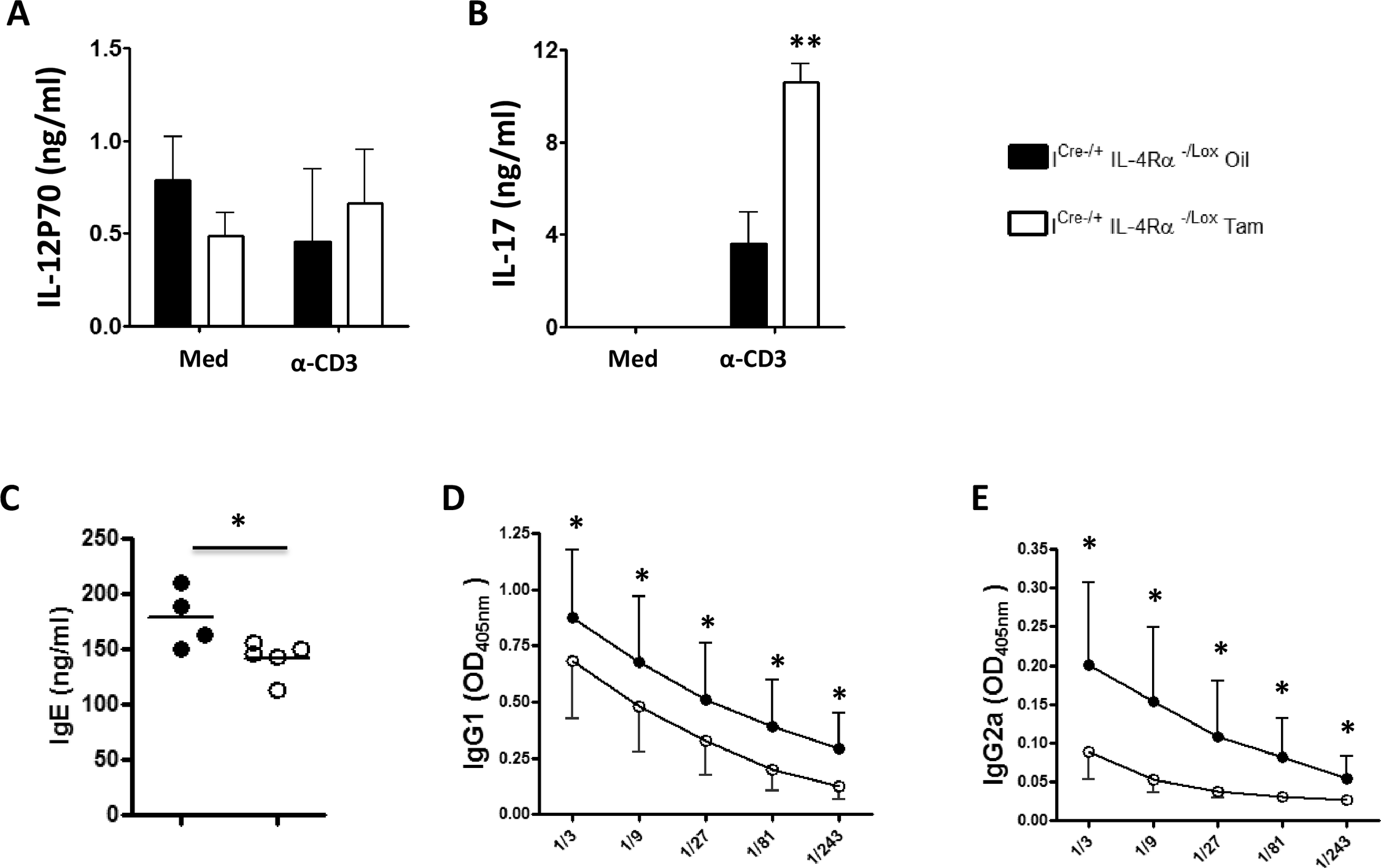

Supplement: S6 Fig — A. IL-12p70 from the supernatant of mdLN cells. B. IL-17 from the supernatant of mdLN cells. C. Serum IgE levels. D. Serum levels of Nb Antigen-specific IgG1 levels. E. Serum levels of Nb Antigen-specific IgG2a levels. (TIF) [file pntd.0005675.s006.tif]
